# Interaction of graphene oxide with tannic acid: computational modeling and toxicity mitigation in *C. elegans*

**DOI:** 10.3762/bjnano.15.105

**Published:** 2024-10-30

**Authors:** Romana Petry, James M de Almeida, Francine Côa, Felipe Crasto de Lima, Diego Stéfani T Martinez, Adalberto Fazzio

**Affiliations:** 1 Brazilian Nanotechnology National Laboratory (LNNano), Brazilian Center for Research in Energy and Materials (CNPEM), Campinas, SP, Brazil; 2 Ilum School of Science, Brazilian Center for Research in Energy and Materials (CNPEM), Campinas, SP, Brazil; 3 Center for Natural and Human Sciences, Federal University of ABC (UFABC), Santo André, 09210-580, São Paulo, Brazil; 4 Center of Nuclear Energy in Agriculture (CENA), University of São Paulo (USP), Piracicaba, SP, Brazil

**Keywords:** biodistribution, density functional theory, ecotoxicity, molecular dynamics, surface interactions, toxicity mitigation

## Abstract

Graphene oxide (GO) undergoes multiple transformations when introduced to biological and environmental media. GO surface favors the adsorption of biomolecules through different types of interaction mechanisms, modulating the biological effects of the material. In this study, we investigated the interaction of GO with tannic acid (TA) and its consequences for GO toxicity. We focused on understanding how TA interacts with GO, its impact on the material surface chemistry, colloidal stability, as well as, toxicity and biodistribution using the *Caenorhabditis elegans* model. Employing computational modeling, including reactive classical molecular dynamics and ab initio calculations, we reveal that TA preferentially binds to the most reactive sites on GO surfaces via the oxygen-containing groups or the carbon matrix; van der Waals interaction forces dominate the binding energy. TA exhibits a dose-dependent mitigating effect on the toxicity of GO, which can be attributed not only to the surface interactions between the molecule and the material but also to the inherent biological properties of TA in *C. elegans*. Our findings contribute to a deeper understanding of GO’s environmental behavior and toxicity and highlight the potential of tannic acid for the synthesis and surface functionalization of graphene-based nanomaterials, offering insights into safer nanotechnology development.

## Introduction

Graphene oxide (GO) has many potential applications in electronics, advanced materials, bio-medicine, energy, agriculture, and environmental technology [1–3]. It consists of a graphene sheet with surface oxygen functional groups such as epoxide, ketone, hydroxy, carboxyl, ether, and carbonyl groups. The sheets present different levels of oxidation as well as specific structures such as edges, wrinkles, and holes. Because of its surface chemistry, GO has better water solubility than graphene; furthermore, it is straightforward to be functionalized and synthesized on larger scales [4]. Nowadays, there is an increasing commercial availability of graphene-related products and companies with large-scale production capabilities of these materials, which includes GO as an intermediate or final product [5–7]. Because of the growing industrial and technological relevance of GO, it is necessary to ensure its safe application, disposal, and regulation. This begins with understanding the behavior of this material in the environment and its impact on living organisms.

Once in a biological/environmental medium, GO undergoes processes such as aggregation, phototransformation, and degradation [8]. Furthermore, because of the presence of sites for different types of interaction mechanisms (i.e., hydrogen bonding, van der Waals interaction, and π–π stacking), its structure favors the adsorption of different molecules (i.e., biomolecules and organic pollutants) and metal ions [8–10]. The physicochemical changes and interactions undergone by GO in the environment greatly influence the biological effects of this material. Recently, Bortolozzo et al. [11] showed that GO degradation by sodium hypochlorite resulted in the mitigation of GO toxicity to *Caenorhabditis elegans*. Ouyang et al. [12] showed that small molecules (e.g., polycyclic aromatic hydrocarbons) and heavy metals, present in the natural water as nanocolloids, potentiate GO’s phytotoxicity. Moreover, biomolecules such as polysaccharides, proteins, lipids, and humic acids may interact with the material’s surface, influencing GO’s colloidal stability, reactivity, and interactions with living organisms. As a consequence, these interactions can lead to diverse effects, ranging from the mitigation of toxicity [12–14] to the enhancement of its toxicity [15–16]. However, microscopic understanding of these processes is missing.

Tannic acid (TA) is an environmentally abundant and commercially available polyphenol with relevant industrial and technological applications [17–20]. TA’s structure comprises five digallic acid units ester-linked to a glucose core. These pyrogallol hydroxy groups participate in hydrogen bonding as well as hydrophobic and electrostatic interactions; also, they are responsible for TA’s high solubility, reactivity to metal cations, binding capacity to molecules and surfaces, and significant reducing and radical scavenging properties [19,21–24]. This range of characteristics made TA attractive to nanomaterial synthesis and functionalization for applications in nanomedicine, sensors, electronics, and composites [25–27]. In these different fields, TA has been applied in green alternative methods of GO synthesis and physicochemical modifications (e.g., reduction and functionalization) [28–30]. In this sense, studying the interaction between TA and GO and the effects on the material toxicity is of technological and environmental relevance.

The nematode *Caenorhabditis elegans* is a well-established in vivo model in human health science and has been considered a promising model in studies of environmental toxicology [31]. Because of its abundance in the environment, its important role in the decomposition and cycling of nutrients, and its sensibility to environmentally relevant concentrations of hazard products, *C. elegans* is considered a good environmental indicator of pollution [32]. Among the advantages of using this organism are growth and rapid reproductive cycle, translucent body, well-known genome, and availability of commercialization of different genetically modified strains [33]. Recent studies of our research group showed that GO presents lethal toxic effects to *C. elegans* at low concentrations (e.g., above 0.1 mg·L^−1^) [11,14]; the main mechanisms of toxicity reported in literature are damage to intestinal cavity and secondary organs, such as reproductive organs and neurons [14,34–35]. The sensibility of the nematode to GO made it a good model to understand how GO’s toxicity changes regarding surface modifications such as interactions with biomolecules.

In this study, we investigate the interaction of GO with TA linked to its impacts on surface chemistry, colloidal stability, lethality, and biodistribution in the *C. elegans* model for the first time. Furthermore, we study in detail TA interactions with GO’s surface employing computational modeling to analyze the interaction mechanisms and GO’s surface modification by TA. The application of in silico methodologies is advantageous in understanding phenomena that cannot be easily accessed experimentally but are useful to predict and interpret experimental results. We performed, therefore, a multilevel study with different theory levels; reactive classical molecular dynamics enabled the exploration of the chemical and conformational changes of TA and GO, whereas ab initio calculations provided information regarding the electronic properties of the system, such as the most reactive sites and their interactions. Our findings provided new insights into toxicity mitigation and behavior of GO in the environment, as well as, the safety of application of TA for synthesis and functionalization of this nanomaterial.

## Results and Discussion

### Experimental characterization

TA is a relevant component of the dissolved organic matter in the environment originating especially from vegetable organic decomposition [17]. Furthermore, because of unique physicochemical properties, TA has been increasingly applied for GO syntheses and surface engineering [29]. Evaluating the changes of GO properties and biological effects after interaction with TA is essential to give us insights into how organic matter affects the behavior and toxicity of this material under real environmental conditions as well as the biological aspects of GO modifications by TA.

To understand the features related to the material’s colloidal behavior, biological effects, and interaction with biomolecules, it is essential to characterize its surface chemistry and dispersion in the medium befitting toxicological studies before and after molecular interactions. The complete characterization of the GO sample is available in [36]. Atomic force microscopy (AFM), Raman spectroscopy, and X-ray photoelectron spectroscopy (XPS) were used to assess size, morphology, number of layers, and surface chemistry of GO. The GO sample used in this study consists of single layers with less than 1.5 nm thickness and a flake size distribution from 18 to 308 nm. The calculated ratio between the intensity of the D (*I*_D_) and G (*I*_G_) bands of Raman is *I*_D_/*I*_G_ = 0.85, indicating that the material has a high number of defects, an indirect indication of oxidation. The surface chemical composition analyzed by X-ray photoelectron spectroscopy (XPS) is 68% of carbon and 32% of oxygen. The functional groups and bonds of carbon are distributed among epoxy/hydroxy (C–O) (52%), carboxyl/esters (C=O) (9.4%), and π–π^*^ (4.2%) moieties, besides graphitic/aromatic carbon (C sp^2^) (5.7%) and aliphatic carbon (C sp^3^) (28%). The properties of this material are in accordance with other GO samples used for nanotoxicology and environmental applications. In this work, we characterized the material after interaction with the moderately hard reconstituted water defined by the U.S. Environmental Protection Agency (EPA), herein named EPA medium, in absence and presence of TA.

#### Atomic force microscopy

**Figure 1:**
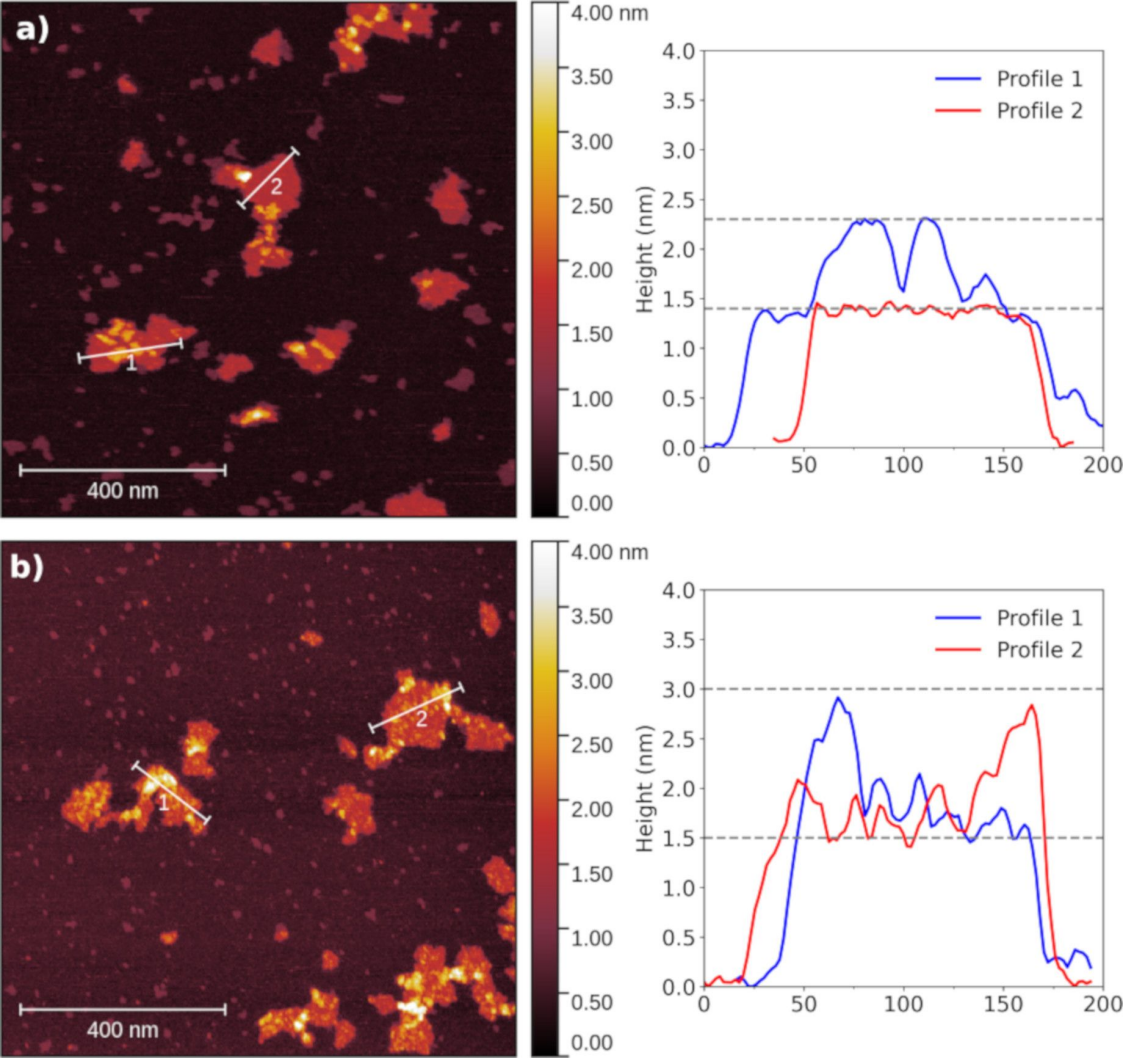
Characterization of the GO and TA interaction system. AFM images of (a) GO and (b) GO incubated with TA (10 mg·L^−1^). The height profile plots on the right present the topology of the marked regions of each sample image.

AFM has been extensively used to characterize the distribution and morphology of biomolecules on the surface of nanomaterials, especially 2D materials [37]. Figure 1a and Figure 1b show AFM images of GO sheets after incubation in EPA medium with and without the addition of TA, respectively. We observed that TA interacts with the GO surface forming a cover up to 3 nm of height, as shown in the height profile analysis. In the absence of TA, GO sheets presented heights from 1.3 nm, indicating single-layer sheets according to data reported in the literature for graphene materials [38], to 2.0 nm in double-layer spots caused by the incubation in the EPA medium.

#### Spectroscopy characterizations

**Figure 2:**
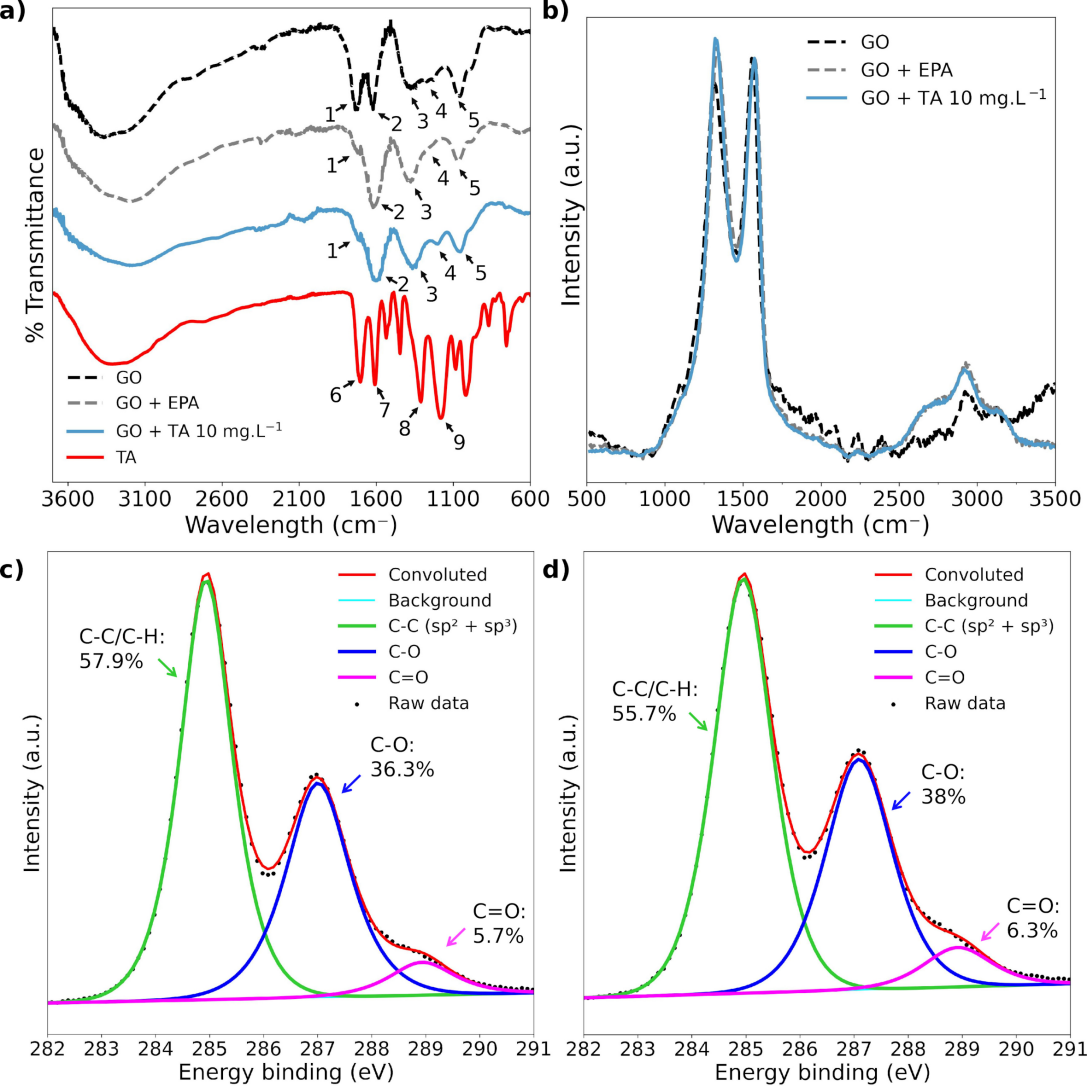
Characterization of GO and TA system. a) FTIR showing absorption signals related to –OH strength band, GO’s fingerprint region with 1734(1), 1625(2), 1390(3), 1230(4), and 1068(5) cm^−1^ bands, and TA-related bands at 1704(6), 1600(7), 1310(8), and 1180(9) cm^−1^; b) Raman spectra normalized by intensity of G band; High-resolution C 1s XPS analysis of c) GO and d) GO with TA (10 mg·L^−1^) showing the peaks of carbon sp^2^+sp^3^ and oxygenated carbon bonds C–O and C=O.

Spectroscopy analysis showed the main chemical groups on the material’s surface, and how their composition changed in the biological medium. In the Fourier-transform infrared spectroscopy (FTIR) analysis (Figure 2a), we observed bands between 3000 and 4000 cm^−1^ related to –OH strength in all spectra. GO spectra presented fingerprint bands at 1734, 1625, 1390, 1230, and 1068 cm^−1^, which correspond to C=O stretching vibrations, aromatic C=C stretching vibrations, C–OH traction, C–O (epoxy) stretching vibrations, and C–O (alkoxy) stretching vibrations, respectively, indicated by the numbers 1 to 5 in Figure 2a [28,39–41]. Important TA bands include 1704, 1600, 1310, and 1180 cm^−1^ (numbers 6 to 9 in Figure 2a), which correspond to C=O, aromatic C=C, phenolic C–OH, and C–O from esters groups connecting the aromatic rings [28,42–44]. Important shifts are observed in the C=O-related band of GO. For TA, this band appears at 1704 cm^−1^ (number 6 in Figure 2a) and for GO at 1734 cm^−1^, while there is a decrease in intensity and a possible blueshift on this band in EPA medium and in the interacting system. The C=C band presented a blueshift from 1625 cm^−1^ to 1610 and 1600 cm^−1^ in the EPA medium and in the presence of TA, respectively. The C–OH band present in GO at 1390 cm^−1^ was shifted to 1360 cm^−1^ after incubation with TA for 24 h. Furthermore, the C–O related bands at 1180 and 1230 cm^−1^ in the spectra of TA and GO, respectively, appeared at 1215 cm^−1^ in the interacting system and had a decreased signal when GO was dispersed in EPA medium in absence of TA. The changes in the vibration energy of these chemical groups indicate that the interactions with TA occur through C=O, C–OH, C–O, and sp^2^ carbon structures present in GO. Such interactions may involve, for example, hydrogen bonds and interactions between π orbitals, which is in agreement with literature regarding humic and tannic acid interactions with GO [45–46]. In the absence of TA, the modulation of the C=0 stretching vibration intensity may indicate coordination of the divalent metal ions Ca^2+^ and Mg^2+^ present in EPA medium [47]. The intensity ratio between *I*_D_ and *I*_G_ bands in Raman spectroscopy analysis ranges from 0.94 ± 0.01, for the GO sample, to 1.02 ± 0.01 and 1.05 ± 0.005 when the material was incubated in EPA medium without and with TA, respectively (Figure 2b). All Raman spectra were normalized to the intensity of the respective G bands. X-ray photoelectron spectroscopy (XPS) presented the composition of GO surface in the presence of TA. XPS survey data suggest that GO after 24 h in EPA medium is composed of 75.33 ± 0.40% carbon and 24.67 ± 0.40% of oxygen, whereas GO after interaction with TA presents 73.30 ± 0.40% of carbon and 26.70 ± 0.44% of oxygen. High-resolution C 1s XPS analysis showed a C–C/C–H peak contribution of 57.96% ± 0.13% to GO in EPA medium and 55.68% ± 1.26% when TA interacts with GO. The oxygenated peaks were 36.35% ± 0.22% (C–O) and 5.69% ± 0.11% (C=O) for GO in EPA medium and 38.03% ± 1.26% (C–O) and 6.28% ± 0.01% (C=O) after TA interaction. Thus, spectroscopy analysis showed no significant changes in GO surface composition after interaction with TA.

#### Colloidal Stability

The study of the colloidal behavior of the material in relevant biological media (regarding, e.g., salinity, pH, or biomolecules) is essential to understand its toxicological outcomes since the aggregation state of this material directly affects delivered dose, internalization, and biodistribution in organisms. In the EPA medium, GO exhibited aggregation and precipitation at concentrations of 5.0 and 10 mg·L^−1^, respectively, a phenomenon attributable to the screening effect of salt ions diminishing the repulsive forces between GO sheets. TA did not improve the stability of these samples. After the 24 h, only the suspensions of 1 mg·L^−1^ of GO did not exhibit visual precipitation (Supporting Information File 1, Figure S1a). The results of dynamic light scattering (DLS) measurements presented in Table S1 (Supporting Information File 1) confirm the aggregation and the subsequent precipitation of GO in the EPA medium; it is noticeable that hydrodynamic diameters rapidly increase in this medium. Although higher TA concentrations slow down aggregation and lead to smaller hydrodynamic diameters after 3 h, after 24 h the samples were completed aggregated with a high polydispersity index. The quality criteria of DLS analysis for GO with a concentration lower than 10 mg·L^−1^ were not satisfactory; therefore, they could not be used to evaluate the dispersion state of more diluted GO suspensions, such as 1 mg·L^−1^ of nanomaterial. However, it is well known that in more diluted suspensions, nanomaterials tend to present better dispersibility, and it is expected that GO remains stable in EPA medium for a longer time.

### Computational simulation of GO–TA interactions

To analyze the surface modification of GO by TA and gain insights into the mechanisms of toxicity mitigation, we employed a computational workflow that involved studying the interactions between GO and TA at different theoretical levels. Molecular dynamics (MD) simulations were performed using the ReaxFF reactive force field to examine the evolution of TA conformation on the surface of a GO flake in an aqueous environment. This allowed us to explore the chemical and conformational changes occurring in TA and GO. Additionally, ab initio calculations were conducted to investigate the electronic properties of the system, including the identification of the most reactive sites on GO, as well as an understanding of how the environment and interactions impact these properties. The combined approach of MD and ab initio calculations provided comprehensive insights into the surface modification process and the underlying mechanisms involved in the interactions between GO and TA.

**Figure 3:**
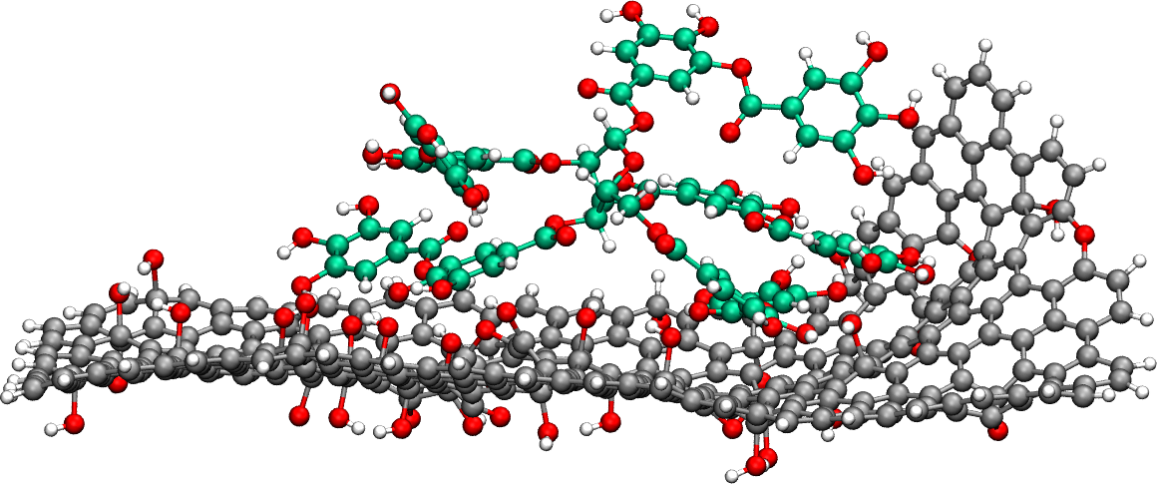
Snapshot of TA on the GO surface obtained from NPT MD at 300 K, parameterized with the ReaxFF reactive force field. The molecular structure view was generated with the VMD software developed with NIH support by the Theoretical and Computational Biophysics group at the Beckman Institute, University of Illinois at Urbana-Champaign (http://www.ks.uiuc.edu/) [48]. This content is not subject to CC BY 4.0.

The MD simulations were performed with TA initially placed at five different sites of GO flakes, namely, the center and the four edges, with the closest atoms at at approximately 2 Å from the sheet. The four edges of the flake differ regarding the carbon configurations (i.e., zigzag or armchair) and defects, one of the armchair edges presents hydroxy groups and one of the zigzag edges presents a broken epoxy site. Figure 3 presents the dynamics of a representative configuration of TA interacting with GO flake. Comparing the evolution of the TA’s configurations in the different simulations, the molecule interacted preferentially with oxygenated groups of GO and with armchair edges rather than zigzag edges. Regarding the latter, the TA molecule moved from the zigzag edge to the armchair edge or even moved away from the GO sheet. We split NPT trajectories into equally spaced snapshots to analyze the TA conformations on the GO surface and to calculate the adsorption energy of TA with density functional theory (DFT). Most interactions between TA and GO occurred through the oxygenated defects in the middle of the sheet and TA oxygen functional groups, as shown in Figure 3. However, it is also possible to identify interactions between these groups and GO’s carbon structure and between carbon atoms of both structures. Furthermore, we analyzed the maximum heights of TA-plus-GO conformations among the snapshots. The values range from 1.5 to 3.0 nm, which corroborates with AFM topography results and indicates that TA mostly forms a single layer of stronger interacting molecules close to the surface.

**Figure 4:**
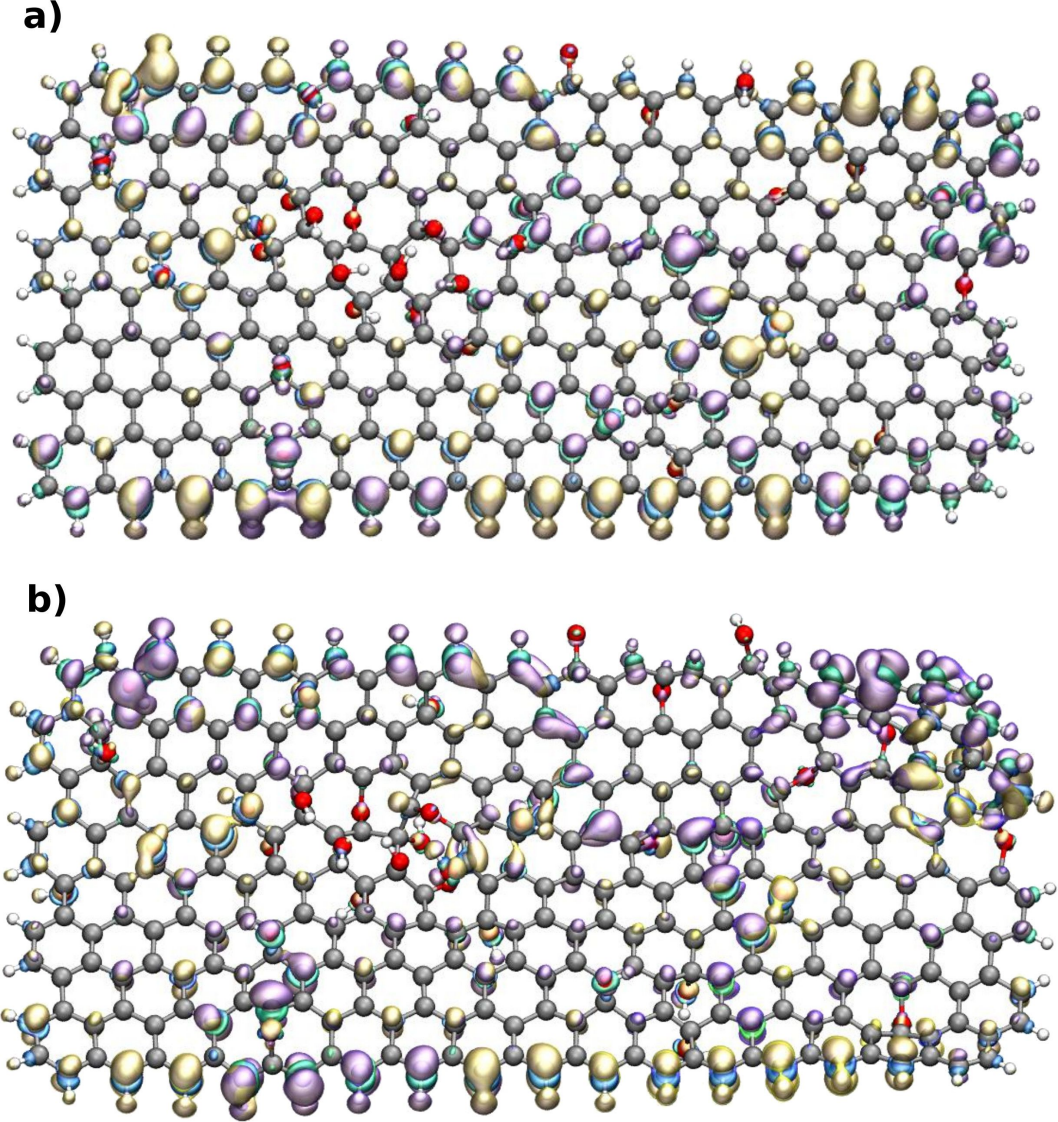
Reactive sites of GO (a) before and (b) after NPT dynamics in aqueous environment. Fukui functions *f*^+^ in yellow (positive) and blue (negative), *f*^−^ in purple (positive) and green (negative). Isosurface of 1 × 10^−3^ *e*/Å^3^. The molecular structure view was generated with the VMD software developed with NIH support by the Theoretical and Computational Biophysics group at the Beckman Institute, University of Illinois at Urbana-Champaign (http://www.ks.uiuc.edu/) [48]. This content is not subject to CC BY 4.0.

DFT calculations allowed us to evaluate the electronic and reactivity properties of the system TA and GO. Fukui functions are a concept used to study the local reactivity of molecules/materials. They provide information regarding how the loss or gain of electrons affects the spatial electronic density of the atoms [49–50], revealing the most reactive sites of the system. We applied Fukui functions to assess the most reactive sites of GO in its initial configuration and after evolution of the sheet configuration in water without TA. Figure 4a and Figure 4b show the charge density plot of the functions *f*^+^ and *f*^−^ of GO before and after NPT MD simulation in an aqueous environment at 300 K. We observed an augmentation of sheet folding and the occurrence of broken bonds, which increased the reactivity of the central oxygenated groups in the flake, where the TA molecule showed preferential interaction in the trajectories.

The adsorption energy of TA on the GO surface ranges from −1.55 to 0.35 eV, with a mean binding energy of *E*_B_ = −0.49 ± 0.08 eV. By selecting the snapshot with the minimum adsorption energy, we calculated the charge transfer of the system using Bader charge analysis, which was 0.1*e*^−^ from GO to TA. The low value of charge transfer indicates that van der Waals (vdW) interaction forces dominate the binding between GO and TA. This is confirmed by the unfavorable binding energy (i.e., positive values up to +2 eV) obtained from DFT calculations when dispersion corrections are not applied. The adsorption energy value is determined by the number and types of interactions involved, such as hydrogen bonds, as well as carbon–carbon and carbon–hydrogen interactions. Supporting Information File 1, Figure S2 shows that the number of interacting atoms (i.e., atoms with distances less than 3.0 Å) between TA and GO is not directly correlated with the binding energy. However, a higher number of weak vdW interactions can lead to similar binding energies as those of snapshots that have fewer interacting atoms but a higher number of hydrogen–oxygen interactions.

**Figure 5:**
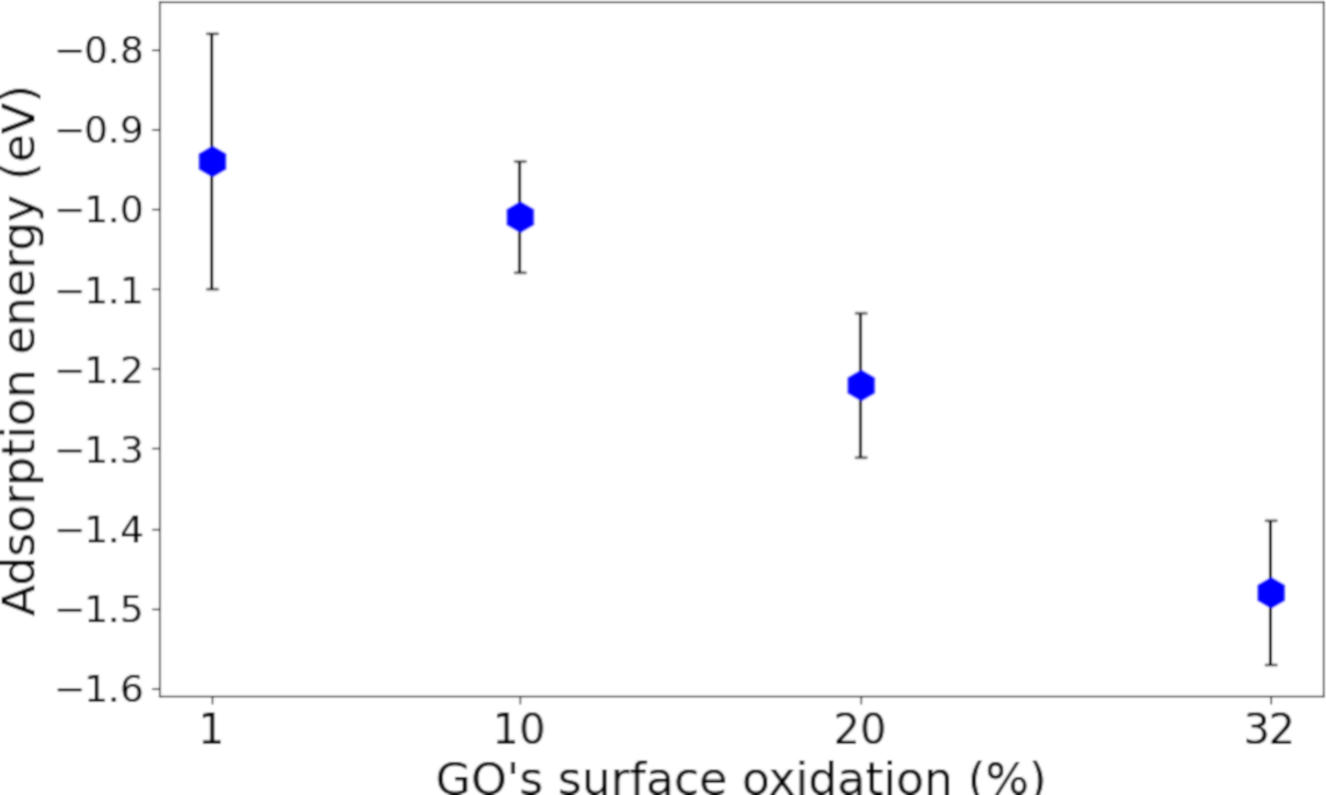
Adsorption energy of TA on GO surfaces with different oxidation degree. The error bars indicate the standard error of the mean from up to ten configurations.

To evaluate the influence of the GO surface’s degree of oxidation on the TA adsorption, we performed MD simulations of TA interactions on periodic GO sheets with oxidation degrees ranging from 1% to 32%. The NPT trajectories were split into equally spaced snapshots, and the average binding energies and standard error of the mean between TA and GO structures were calculated from DFT calculations. Figure 5 shows that the interaction between TA and GO increases with the oxidation level of the GO surface, which can be explained by the increased number of functional groups that participate in stronger van der Waals interactions (Supporting Information File 1, Figure S3).

### Biological effects in *C. elegans*

*C. elegans* has been considered a relevant in vivo model for nanomaterials toxicity and ecotoxicity. Several works demonstrate that this organism shows sensibility to GO in low doses. In previous works, our research group found that GO decreased nematode survival at concentrations above 0.1 mg·L^−1^ [11,14]. GO potentially affects the intestinal cavity and secondary organs of *C. elegans*. The intestine is the primary organ to be exposed to ingested hazardous substances or materials and plays an important role in protecting other organs. Different studies show an increased intestinal permeability after exposure to GO, enabling the material to reach adjacent organs such as the gonads [14,51–52]. Wu et al. [51] found that prolonged exposure to GO causes significant damage to intestinal microvilli cells . Furthermore, Dou et al. [53] showed that GO triggers cell autophagy as a protective response to the material. Apoptosis was observed in germline cells, indicating that GO can damage gonad development and reduce the reproduction rate of *C. elegans* [35,54]. Oxidative stress is one of the central mechanisms and, in fact, the main cause of the toxicity outcomes discussed above. It is associated to changes in the function or expression of superoxide dismutase, “Rieske” iron-sulfur protein, mitochondrial complex I, and the ubiquinone biosynthesis protein COQ7 [51,53–55]. The co-exposure of GO with antioxidant molecules, such as ʟ-cysteine and ascorbate, can mitigate the oxidative effects of the material and minimize GO’s toxicity [35,53]. Moreover, GO also shows important neuronal effects; for example, it influences protein–protein binding in the organism, activating or suppressing neuronal receptors and influencing the neurotransmission process in *C. elegans* [34–35,56].

GO’s toxicity is highly related to its surface chemistry; changes of the functional groups of the surface impact its biological effects. Yang et al. [57] showed that changes in the oxygen content of GO may improve its biocompatibility. They found that GO sheets with reduced oxygen content and relatively more –COOH groups did not presented the common GO toxicity effects to *C. elegans*, such as increased intestinal permeability, microvilli damage, material translocation to other organs or oxidative stress. Similarly, Rive et al. [58] did not detect any detrimental effects in *C. elegans* exposed to amino-functionalized GO. Moreover, biomolecules interacting with the GO surface also have an effect on its toxicity, Côa et al. [14] observed that a bovine serum albumin corona mitigated the acute toxicity of GO, although it did not fully suppress long-term effects such as reproductive toxicity.

#### Acute toxicity

In this work, we found that the lowest GO concentration that caused significative effects on survival was 1.0 mg·L^−1^, with a mortality of approximately 30%. Concentrations of 5.0 and 10 mg·L^−1^ of GO yielded similar mortality rates, up to 40% of mortality, which may be an effect of aggregation and precipitation of the material in the test medium. The colloidal instability of the nanomaterial in the test medium impacts the dose bioavailable to *C. elegans*, which stays on the well’s bottom most of the time. At 5 mg·L^−1^, GO aggregates and precipitates in EPA medium, which increases the exposure to *C. elegans*. The amount of material ingested by the nematode is limited by the size of its mouth, which is where most of the uptake occurs. *C. elegans* exhibits a size-selective feeding mechanism, which transports particles in the size range from 0.5 to 3 μm to the intestinal lumen [59–60]. Therefore, even at higher doses, we did not observe a linear relationship between *C. elegans*’ survival and the material’s concentration.

**Figure 6:**
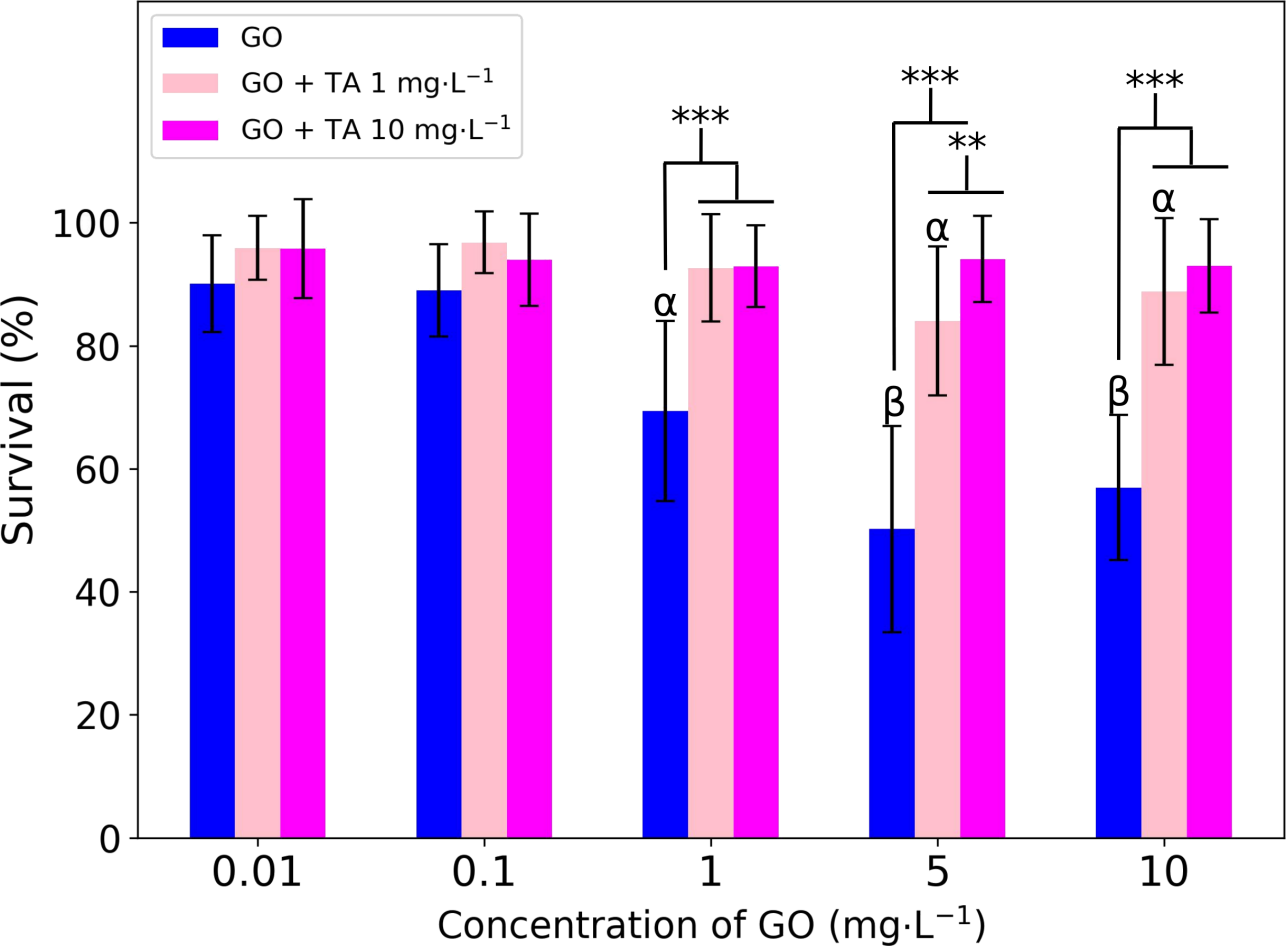
Effects of GO in presence or absence of TA on *C. elegans’* survival. α and β indicate survival rates significantly different from the control (100% of survival) with *p* ≤ 0.05 (one-way ANOVA). *** and ** indicate difference in the treatments with *p* ≤ 0.001 and *p* ≤ 0.05 (two-way ANOVA), respectively. The error bars are calculated from 16 to 18 data points on survival.

Considering this, we evaluated the effects of tannic acid on the GO toxicity in a co-exposition system. The survival rates of *C. elegans* at GO concentrations ranging from 0.0001 to 10 mg·L^−1^ were analyzed in the presence of 1 and 10 mg·L^−1^ of TA. Figure 6 shows the survival rates of *C. elegans* after exposition to only GO and to GO in the presence of TA. We observed a dose-dependent mitigation effect of TA. A concentration of 1 mg·L^−1^ TA raised the lowest observed adverse effect level of GO to 5 mg·L^−1^; 10 mg·L^−1^ of TA completely mitigated the acute effects of GO under the conditions tested.

#### Biodistribution study

**Figure 7:**
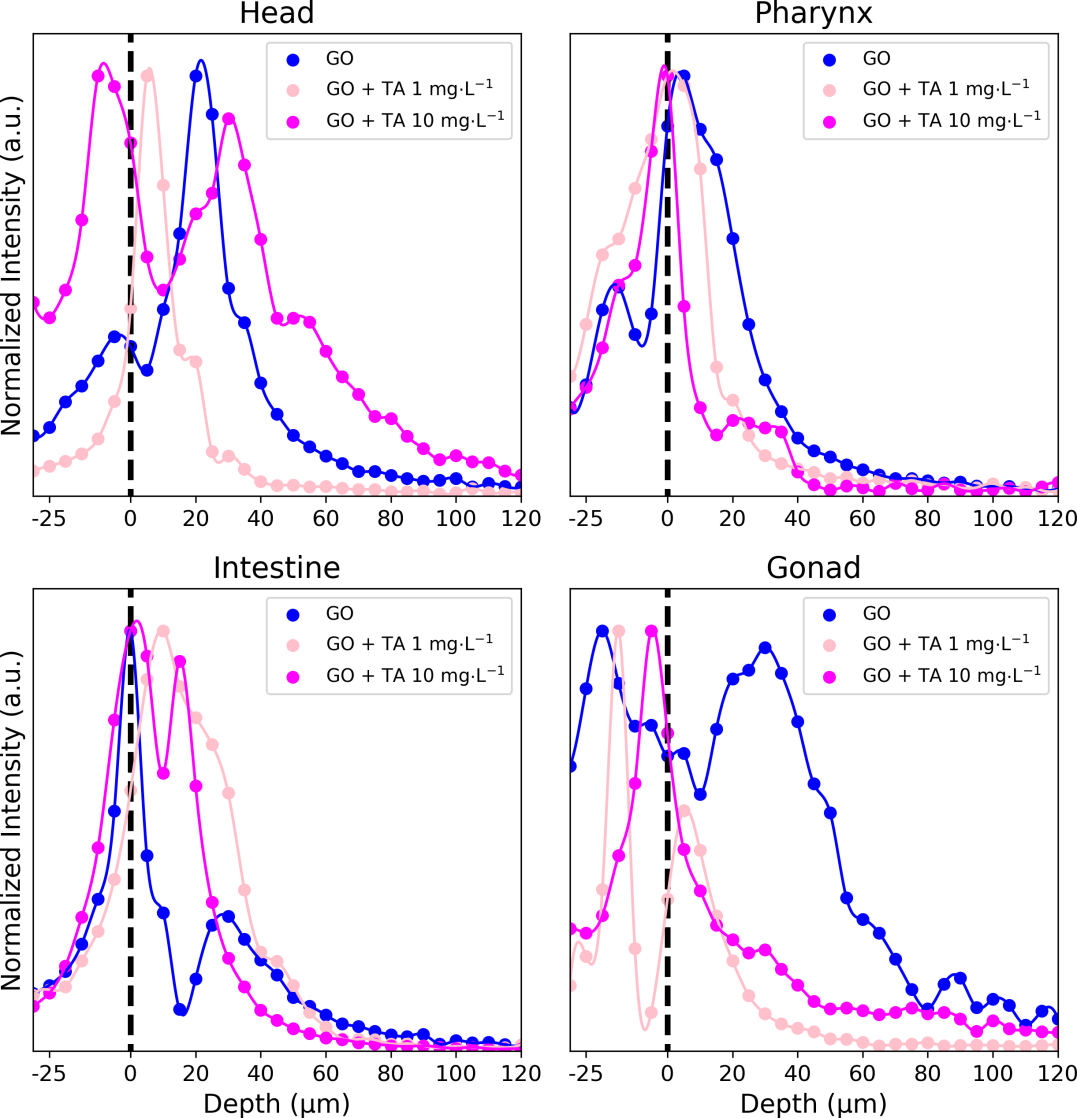
G-band intensity depth profiles (from −30 to 120 μm) used to monitor biodistribution of GO, and GO with 1 and 10 mg·L^−1^ of TA in different tissues of nematodes.

Confocal Raman spectroscopy analyses were conducted to evaluate the effects of TA on the biodistribution of GO in nematode tissues. The unique signature of GO’s Raman spectra, with the two distinct D (≈1300 cm^−1^) and G (≈1600 cm^−1^) bands, enables the localization and identification of the material in biological tissues. Depth profile measurements were performed in the head, pharynx, intestine, gonad, and egg regions. At each point, the upper cuticle was considered as the distance 0 μm, and to differentiate GO’s internal and external signals, Raman spectra were acquired from −30 to 120 μm, with steps of 5 µm. The intensity of the G band at each depth was recorded in the profiles shown in Figure 7, which were normalized regarding the maximum intensity found in the region. The intensity profiles and the respective spectra, were used to draw conclusions about GO’s internalization in the organisms. According to Figure 7, GO was found along the entire nematode cuticle. Furthermore, GO was found internally in the head, intestine, and pharynx of nematodes, regardless of the presence of TA. Internalization of GO in the gonads was also observed and to some extend in eggs, although in the latter the occurrence of GO signal decreased after the addition of TA.

Raman analysis showed that TA does not affect the biodistribution of GO in *C. elegans*, including in secondary organs, although it changed the mortality caused by the material. Experimental and theoretical characterization show that TA can interact with the GO surface. DFT calculations demonstrated that TA adsorbs at the most reactive sites of GO, which can be related to the decrease of the material’s toxicity by impairing these sites to interact with critical molecules or tissues that initiate acute toxicity pathways. However, because of the translocation of GO to different organs in the presence of TA, GO still might cause long-term effects, which need to be subject of further investigations. Concomitantly, it is known that the polyphenols such as TA exhibit properties that are beneficial to health, such as antimicrobial, anti-inflammatory, and antioxidant capacities [61–62]. Saul et al. [63] showed that different polyphenols have life-prolonging and stress-reducing properties to *C. elegans*. Up to 300 μM (≈500 mg·L^−1^) TA promotes longevity in *C. elegans*, which is called hormesis effect; at higher concentrations, TA is actually toxic [64]. TA exposure induces different resistance mechanisms against pathogens, heating stress, and oxidative stress, which may increase the resistance against the hazardous effects of GO. TA upregulates natural protective pathways against oxidative stress, increasing the expression of antioxidant systems such as reduced glutathione, superoxide dismutase, and catalase [61]. Besides that, the metal chelating properties of TA may influence oxidative pathways dependent of these cofactors, such as Fenton’s reaction and copper-mediated formation of free radicals. TA may also act as direct radical scavenger in these reactions [65–68]. Moreover, TA exhibits an antinutritional effect and may induce the calorie restriction (CR) pathway in *C. elegans*, which is a potential cause of the TA-mediated lifespan extension [63–64]. The CR effect could decrease the acute toxicity effects of GO by decreasing the ingestion of the material by *C. elegans*.

## Conclusion

Assessing the effects of TA on GO toxicity, we gained insights on how components in environmental media, such as organic matter, modulates the biological effects of GO, which are still not entirely understood. Experimental and theoretical analyses have demonstrated that TA interacts with GO surfaces via oxygen-containing functional groups, resulting in enhanced binding energies. Nevertheless, the adsorption of TA also involves weaker interactions mediated by the carbon framework. DFT calculations using Fukui functions demonstrated that TA interacts with the most reactive sites of GO, and van der Waals interaction forces dominate the binding energy. We observe a dose-dependent mitigation effect of TA on the toxicity of GO in the model *C. elegans*. TA at a concentration of 1 mg·L^−1^ raised the lowest concentration of GO affecting the survival of *C. elegans* to 5 mg·L^−1^; at 10 mg·L^−1^, it mitigated completely the mortality effects of GO under the tested conditions. TA did not alter the biodistribution of GO in the intestinal lumen, head, gonads, and eggs of the nematodes. Possible mechanisms for the reduced toxicity are (i) hindering of reactive sites of the GO surface from interactions with molecules or tissues that play a role in the toxicity pathways, (ii) TA-induced stress resistance mechanisms in *C. elegans* alleviating the effects of GO’s acute toxicity, such as oxidative stress, and (iii) TA acting directly as antioxidant or chelating cofactor in oxidative pathways in *C. elegans*. Further experimental analysis should be carried out to evaluate the effects of TA on the long-term toxicity effects of GO and confirm the TA mitigation mechanisms. This work contributes towards a more realistic view of GO toxicity and fate under environmental conditions. Furthermore, it highlights the potential of TA in surface engineering of graphene-based nanomaterials.

## Methods

### Materials

GO was synthesized via chemical exfoliation of graphite by modified Hummers method [69] according to [70]. Graphite (5.0 g) and NaNO_3_ (3.75 mg) are added to a reaction flask in a bath of ice and covered with concentrated H_2_SO_4_ (370 mL). The mixture is stirred for 20 min, then KMnO_4_ (22.5 mg) is added gradually over 1 h. The reaction is kept under stirring for 72 h at room temperature, and then it is diluted with 300 mL of deionized water and kept for another hour at 95 °C. The temperature is then reduced to 60 °C, and H_2_O_2_ (15 mL, 30% w/w) is added to complete the oxidation of graphite and the reduction of residual KMnO_4_; the mixture is left under stirring overnight. At the end, the material is precipitated by centrifugation and washed with H_2_SO_4_ (3.0%) and H_2_O_2_ (0.5%) to remove residues of oxidants and inorganic impurities. The remaining residuals of salts are removed by dialysis in distilled water for approximately three days. The obtained GO suspension is then lyophilized for storage [36].

### Characterization

The physicochemical and colloidal characterization of nanomaterials is essential to their toxicity assessment and biological/environmental application. The properties of the materials in biological environments may differ significantly depending on the composition of the medium (e.g., aggregation state, surface charge, and dissolution) and determine their biological effects.

Therefore, the initial step to assess nanomaterials toxicity is to evaluate their colloidal characteristics. GO stock dispersions (400 mg·L^−1^) were prepared according to OECD Guideline no. 318 [71]. The GO powder (10 mg) was pre-wetted with 1 mL of ultrapure water and left as a wet-paste for 24 h. Then, ultrapure water (25 mL) was added, and the suspension was sonicated in an ultrasonic bath. The sonication time was controlled by analyzing the material’s hydrodynamic diameters by dynamic light scattering (DLS). Dispersion aliquots for measurement were collected every 10 min, and the sonication was performed until there were no significant changes in the hydrodynamic diameter. Both conditions were tested, the time for the first dispersion and for the redispersion of GO. The GO stock suspensions were stored for a maximum of 14 days, as recommended by OECD Guideline no. 318 [71].

The colloidal characteristics of GO were evaluated according to toxicity assay conditions by photographic monitoring and DLS. The behavior of environmental relevant concentrations (10–20 mg·L^−1^) of tannic acid solution in the test medium and the influence on the colloidal stability of GO were also analyzed. The toxicity assays in *Caenorhabditis elegans* were performed in moderately hard reconstituted water defined by the U.S. Environmental Protection Agency (named here as EPA medium), whose composition includes 60.0 mg·L^−1^ CaSO_4_·2H_2_O, 60.0 mg·L^−1^ MgSO_4_, 96.0 mg·L^−1^ NaHCO_3_, and 4.0 mg·L^−1^ KCl. The initial range of GO concentration tested against *C. elegans* was 0.0001 to 10 mg·L^−1^, and the duration of exposure was 24 h for acute toxicity assays. Visual monitoring of the colloidal behavior of GO was performed for a period of 24 h, comparing the stability of 1.0, 5.0, and 10 mg·L^−1^ suspensions of nanomaterial in EPA medium with and without the presence of 10 mg·L^−1^ of tannic acid. A GO suspension of 10 mg·L^−1^ in ultrapure water was used as a control. Furthermore, a 10 mg·L^−1^ TA solution was also observed for this period of time regarding precipitation or possible change of color due to reactions such as oxidation. The colloidal stability of all suspensions with 10 mg·L^−1^ GO, with and without TA, was also monitored by DLS. Furthermore, a new sample, 10 mg·L^−1^ GO and 20 mg·L^−1^ of TA in EPA medium, was monitored in order to test if a higher concentration of TA would improve the colloidal stability of GO.

AFM (MultiMode VIII microscope, Bruker), Raman spectroscopy (XploRA PLUS, Horiba), FTIR spectroscopy (IRSpirit Shimadzu), and XPS (K-Alpha XPS Thermo Fisher Scientific) were used to assess changes in the morphology and surface chemistry of GO while interacting with TA. For AFM analysis, 10 mg·L^−1^ GO was incubated in EPA medium for 24 h with and without 10 mg·L^−1^ TA. Then, to avoid salt interference, the suspension was washed three times with deionized water and dripped on mica substrate. The incubation procedure was repeated for spectroscopy analysis. For Raman and FTIR analysis, the suspensions were dried using the speed-vacuum method at room temperature; for XPS, the suspensions were dripped on a silicon substrate.

### Computational methods

MD simulations of interactions between TA and the GO surface were performed in LAMMPS, applying ReaxFF reactive force field [72]. MD simulations were conducted under constant pressure (*P*) and temperature (*T*), the so-called NPT conditions, for a period of 4.00 ns, with a time step of 0.25 fs, starting from the system in equilibrium at 300 K. The initial system consisted of a representative GO flake obtained from [73], with dimensions of 42 × 20 Å and an oxidation level of 12.5%, and the TA free-energy-minimum conformer calculated in aqueous environment obtained from a previous work [74]. TA was initially placed in five different positions, that is, the center and the four different edges of the GO flake, with the closest atoms at approximately 2 Å distance from the sheet. The simulations were performed in a box of 60 × 60 × 60 Å filled with water molecules to reach a density of 1 g/cm^3^. In order to evaluate the effects of the GO oxidation level on the interactions with TA, we also performed MD simulations with periodic GO sheets with oxidation levels from 1 to 32%, the latter corresponding to the oxidation degree of the samples used in the toxicity assays. Periodic system simulations were performed under NPT conditions for 2.5 ns at 300 K. TA was initially placed at the center of the box at approximately 2 Å distance from the sheet. The box dimensions were approximately 40 × 35 × 40 Å filled with water molecules to reach the density of 1 g/cm^3^.

DFT calculations were performed using VASP [75–76]. The Perdew–Burke–Ernzerhof (PBE) generalized gradient approximation was used for the exchange–correlation term [77]. The kinetic energy cutoff for the plane-wave expansion was 520 eV. Furthermore, the nonlocal van der Waals density functional (vdW-DF) method was applied to account for dispersion interactions [78]. To account for solvation effects, the implicit solvation model developed by Mathew et al. was applied in the calculations [79]. To evaluate reactivity changes, Fukui functions were calculated [49–50,80–82], analyzing differences in electron density when an electron is removed (Equation 1) or added (Equation 2) to the molecule:


[1] $$f^{-}=\rho \left(N_{\text{e}}\right)-\rho \left(N_{\text{e}}-1\right),$$

[2] $$f^{+}=\rho \left(N_{\text{e}}+1\right)-\rho \left(N_{\text{e}}\right),$$

where the electron densities ρ(*N*_e_), ρ(*N*_e_ − 1), and ρ(*N*_e_ + 1) correspond to systems with *N*_e_, *N*_e_ − 1, and *N*_e_ + 1 electrons, respectively.

### Biological assays

Initial toxicity assays were conducted to evaluate the effects of GO on the survival of *C. elegans*. Acute toxicity assays were performed according to the protocol developed by Maurer et al. [83]. The toxicity experiments were conducted in 24-well plates with a total test volume of 1.0 mL per well. Each well contained ≈20 young adult *C. elegans*, that is worms between the stages L2 and L3 of development, approximately 30 h of age, obtained through the synchronization procedure described in [14,84]. The worms were exposed to GO at final concentrations of 0.0001, 0.001, 0.01, 0.1, 1.0, 5.0, and 10 mg·L^−1^ in EPA medium. Furthermore, negative controls were carried out using ultrapure water as the test substance because the GO stock dispersions were prepared in this medium. The nematodes were exposed for 24 h, and live organisms were counted using a stereomicroscope at the end. To evaluate the effect of tannic acid on the GO toxicity, the survival rates of *C. elegans* at GO concentrations ranging from 0.0001 to 10 mg·L^−1^ were also analyzed in the presence of 1 and 10 mg·L^−1^ of TA. Each exposure condition was performed in independent triplicates, with six replicates each. Consequently, each condition yielded between 16 and 18 data points. To assess statistical differences in survival rates, we conducted a one-way ANOVA followed by Dunnett’s multiple comparison post-hoc test to evaluate significance among the GO concentrations and the control, and a two-way ANOVA to determine significance among conditions with and without 1 and 10 mg·L^−1^ TA.

The biodistribution of GO in nematodes was investigated using confocal Raman spectroscopy. Young adult worms were exposed to a concentration of 5 mg·L^−1^ of GO material, both with and without TA, at concentrations of 1 and 10 mg·L^−1^, following the same protocol used in the acute toxicity assays. After 48 h, the nematodes were fixed with 4% paraformaldehyde (Lot #SLBF2268V, Sigma-Aldrich) and washed twice with EPA medium to remove any excess nanomaterial. Raman spectra were obtained from various parts of the nematodes, including the head, pharynx, intestine, gonad, and eggs. To differentiate between internal and external signals of GO, depth profiles ranging from −30 to 120 μm (assuming 0 μm as the upper cuticle) were acquired at each position, with steps of 5 µm [85–86]. Raman spectra were acquired using a confocal Raman spectrometer equipped with an optical confocal microscope (50× objective). The excitation wavelength was set at 532 nm, and spectra were acquired with five accumulations of 5 s each. The slit width was set to 50 μm, and the hole width was set to 100 μm, resulting in a laser spot of approximately 1 µm on the sample.

## Supporting Information

File 1 Supplementary material.

## Data Availability

The data that supports the findings of this study is available from the corresponding author upon reasonable request.
