# Supplementary material for: Interaction of graphene oxide with tannic acid: computational modeling and toxicity mitigation in C. elegans
Source: Beilstein J Nanotechnol. 2024 Oct 30;15:1297–311. doi: 10.3762/bjnano.15.105 (PMC11533115; doi:10.3762/bjnano.15.105)
Supplement: File 1 — Supplementary material. [file Beilstein_J_Nanotechnol-15-1297-s001.pdf]

BEILSTEIN JOURNAL OF NANOTECHNOLOGY

Supporting Information

for

Interaction of graphene oxide with tannic acid: computational modeling and toxicity mitigation in *C. elegans*

Romana Petry, James M. de Almeida, Francine Côa, Felipe Crasto de Lima, Diego Stéfani T. Martinez and Adalberto Fazzio

Beilstein J. Nanotechnol. **2024,** *15,* 1297–1311. doi:10.3762/bjnano.15.105

Supplementary material

License and Terms: This is a supporting information file under the terms of the Creative Commons Attribution License (https://creativecommons.org/licenses/by/4.0). Please note that the reuse, redistribution and reproduction in particular requires that the author(s) and source are credited and that individual graphics may be subject to special legal provisions.
The license is subject to the *Beilstein Journal of Nanotechnology* terms and conditions: (https://www.beilstein-journals.org/bjnano/terms)

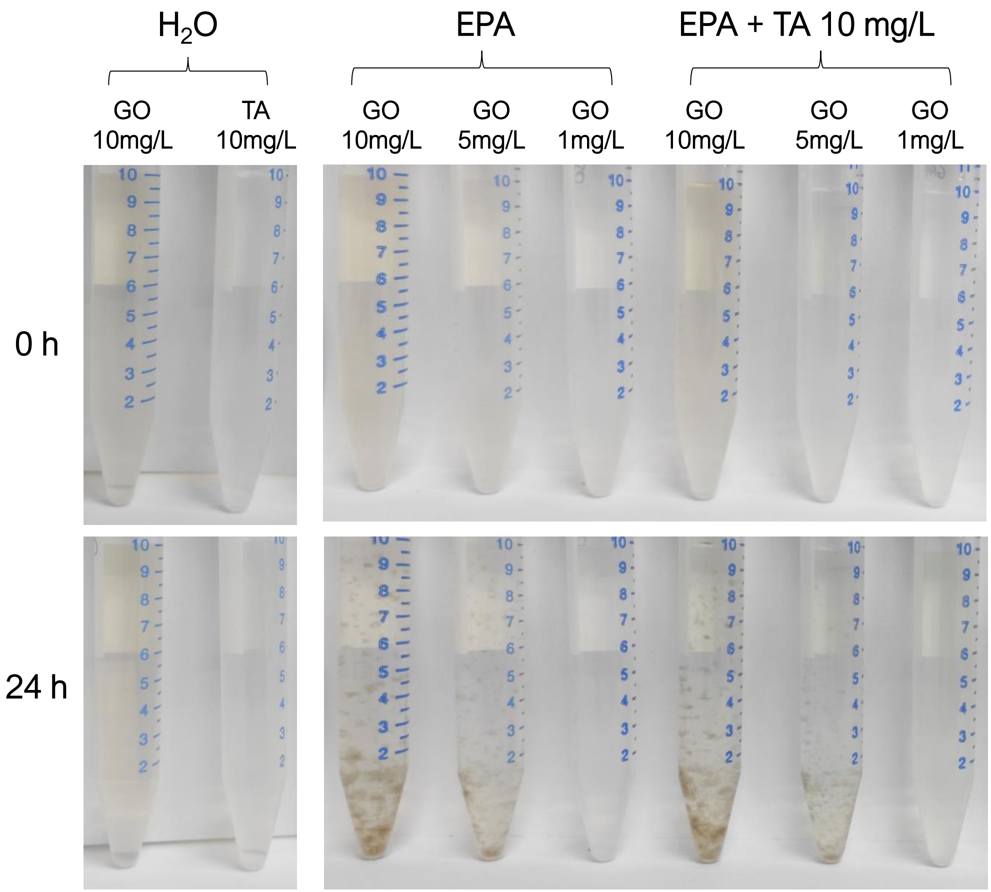

Figure S1: Colloidal stability assay of 1.0, 5.0 and 10 mg·L^{-1} GO suspensions in EPA medium with and without the presence of 10 mg·L^{-1} of tannic acid. The GO suspension of 10 mg·L^{-1} in ultrapure water is used as a stable control. Also, a 10 mg·L^{-1} TA solution was monitored for precipitation or change of color.

Table S1: Monitoring of colloidal stability by DLS of 10 mg·L^{-1} GO suspensions in EPA medium with and without the presence of 10 mg·L^{-1} or 20 mg·L^{-1} of tannic acid. HD: hydrodynamic diameter, PDI: polydispersivity index, TA10: 10 mg·L^{-1} of tannic acid, TA20: 20 mg·L^{-1} of tannic acid.

	**0 hours**		**3 hours**		**24 hours**	
**Sample**	**HD (nm)**	**PDI**	**HD (nm)**	**PDI**	**HD (nm)**	**PDI**
**GO+H2O**	86.4 ± 3.7	0.245	84.7 ± 3.8	0.252	85.3 ± 2.3	0.251
**GO+EPA**	182 ± 15.6	0.314	2313.2 ± 248.7	0.954	3811.2 ± 1064	1.429
**GO+TA10+EPA**	115.3 ± 20.3	0.284	1675.4 ± 208.8	0.812	3421.2 ± 733.0	1.403
**GO+TA20+EPA**	149.9 ± 35.4	0.338	552.1 ± 196.2	0.585	2574.7 ± 681.5	1.190

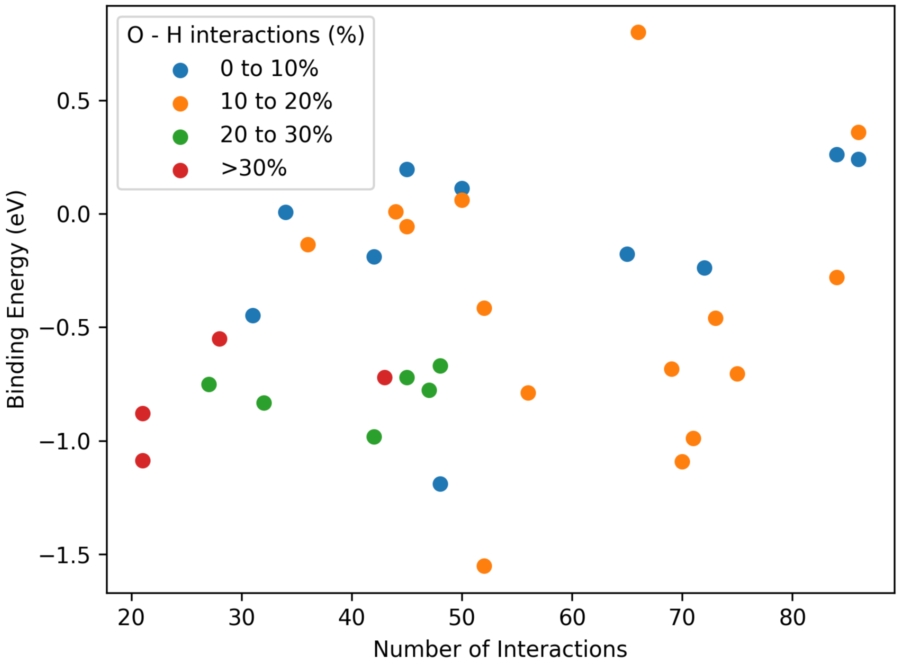

Figure S2: Relation between binding energy and the number of interacting atoms in the interface of GO and TA. The colors represent the percentage of interactions that occur between O and H atoms.

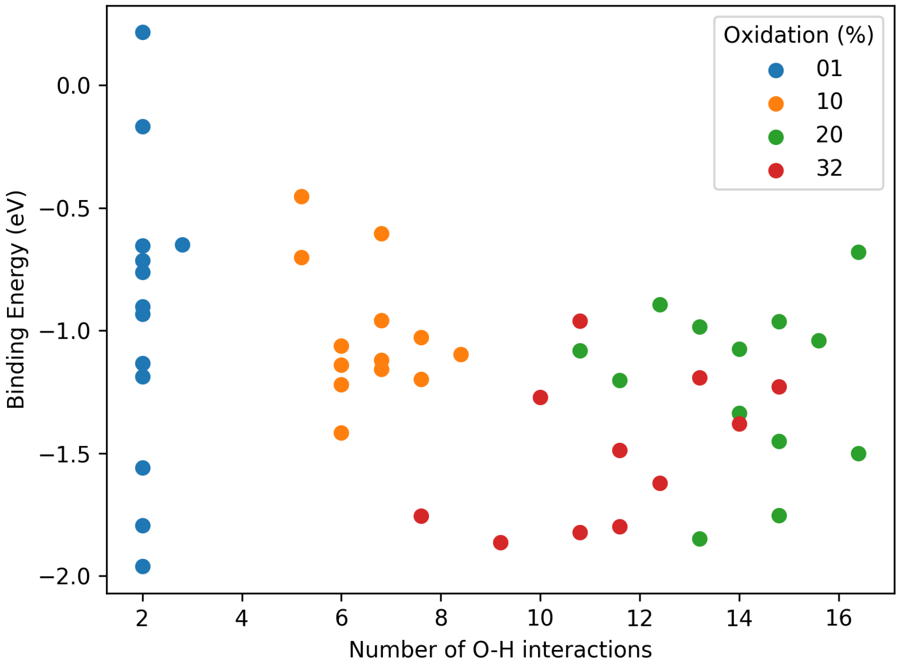

Figure S3: Relation between binding energy and the number of O-H interacting atoms in the interface of GO and TA. The colors represent GO's surface oxidation.
